# A Thiosemicarbazone Derivative as a Booster in Photodynamic Therapy—A Way to Improve the Therapeutic Effect

**DOI:** 10.3390/ijms232315370

**Published:** 2022-12-06

**Authors:** Robert Gawecki, Jaroslaw Polanski, Anna Mrozek-Wilczkiewicz

**Affiliations:** 1 A. Chełkowski Institute of Physics, University of Silesia in Katowice, 75 Pułku Piechoty 1a, 41-500 Chorzow, Poland; 2 Institute of Chemistry, University of Silesia, 40-007 Katowice, Poland

**Keywords:** thiosemicarbazone, iron chelators, ABC transporters, photodynamic therapy, phototoxic effect, heme

## Abstract

Photodynamic therapy is one of the most patient friendly and promising anticancer therapies. The active ingredient is irradiated protoporphyrin IX, which is produced in the body that transfers energy to the oxygen-triggering phototoxic reaction. This effect could be enhanced by using iron chelators, which inhibit the final step of heme biosynthesis, thereby increasing the protoporphyrin IX concentration. In the presented work, we studied thiosemicarbazone derivative, which is a universal enhancer of the phototoxic effect. We examined several genes that are involved in the transport of the heme substrates and heme itself. The results indicate that despite an elevated level of ABCG2, which is responsible for the PpIX efflux, its concentration in a cell is sufficient to trigger a photodynamic reaction. This effect was not observed for 5-ALA alone. The analyzed cell lines differed in the scale of the effect and a correlation with the PpIX accumulation was observed. Additionally, an increased activation of the iron transporter MFNR1 was also detected, which indicated that the regulation of iron transport is essential in PDT.

## 1. Introduction

Generally, cancer therapies have the most side effects of any of the therapies that are used today. These side effects often create serious psychological and medical problems. Therefore, therapies that are safe for the patient should be developed. One such therapy is photodynamic therapy (PDT), which is one of the few anticancer methods that is relatively safe for the patient [1,2,3]. The reason for its low toxicity is its selectivity, which is associated with its local effect. In PDT, the photosensitizer, which can be called the active ingredient, accumulates in the tumor and triggers a toxic effect there [4]. The specificity of the therapy itself lies in the interaction of oxygen, the light of the appropriate wavelength and the photosensitizer [5]. An additional advantage of ALA-PDT therapy is that the photosensitizer is produced in the body. As a prodrug, 5-ALA is a substrate for the production of protoporphyrin IX (PpIX), which absorbs the energy from light and then transmits that energy to oxygen, which in turn leads to the production of free radicals and singlet oxygen [6]. The natural synthesis of PpIX has its limitations, however, because PpIX is not the final product. PpIX is the penultimate link in the production of heme, which plays a huge role in our body, but is not a photosensitizer. Therefore, in ALA-PDT therapy, iron chelators that inhibit the incorporation of iron ions into the porphyrin ring are often used [7]. Despite extensive research on ALA-PDT, there are still many unanswered questions, such as the mechanism by which selective PpIX accumulates in cancer cells, and why various cell lines have differing abilities to accumulate PpIX or respond to irradiation. Even less is known about the mechanisms of action of ALA-PDT in combination with iron chelators.

**Figure 1:**
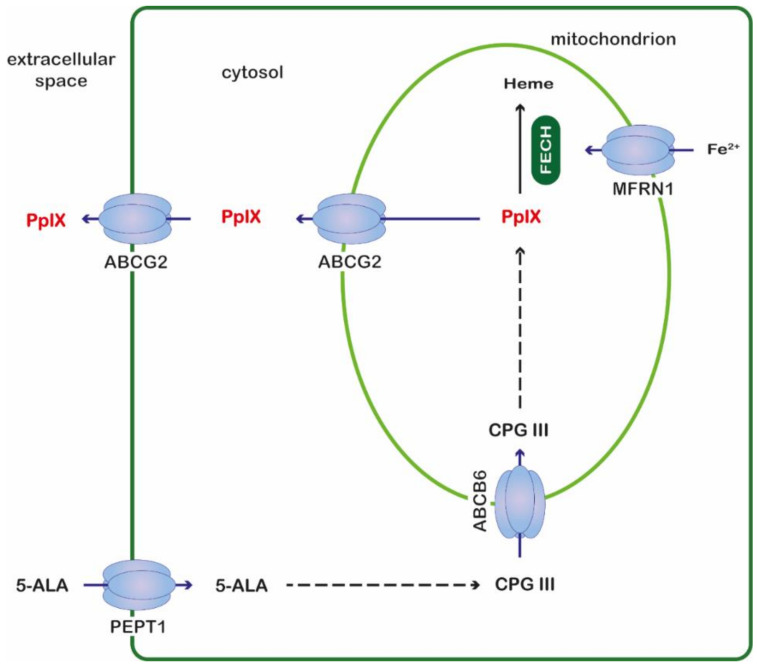
Schematic representation of selected transporters involved in the transport of intermediates of the heme biosynthetic pathway. ABCB6—ATP-binding cassette subfamily B member 6, ABCG2—ATP-binding cassette subfamily G member 2, 5-ALA—5-aminolevulinic acid, CPG III—coproporphyrinogen III, FECH—ferrochelatase, MFRN1—mitoferrin 1, PEPT1—peptide transporter 1, PpIX—protoporphyrin IX.

In this work, we continued the study of the use of iron chelators from the TSC group to improve ALA-PDT therapy. In our previous work, we selected a thiosemicarbazone derivative with quinoline and thiomorpholine fragments; namely, derivative **2** [8]. Because this compound boosts the PpIX concentration in several cell lines, it is the most universal. Therefore, we continued the research with derivative **2** and focused on the transporters that are involved in the heme biosynthesis pathway intermediates: flux and the phototoxic effects (Figure 1). We continued the study on four cancer cell lines (Mcf-7, HCT 116, Hs683 and A549) because we observed interesting differences in the PpIX accumulation and the expression of the genes that are involved in heme biosynthesis and the degradation pathway in untreated cells, and in cells that had been treated with 5-ALA or 5-ALA with TSC. The Hs683 cell lines especially exhibited an atypical behavior that has not yet been described in the literature. In the current work, we extended the study to several targets that are responsible for the transport of the substrates for heme into a cell and its efflux from a cell.

The first analyzed gene was the peptide transporter 1 (PepT1), which plays a crucial role in the delivery of a prodrug (5-ALA) to a cell [9,10]. PepT1 is a prototype transporter of the SLC15 family [11] and its induction is connected with an enhanced therapeutic effect via an increased transport of 5-ALA into a cell [12]. A similar situation was discovered for the ATP-binding cassette (ABC) transporter ABCB6, which transports coproporphyrinogen III into the mitochondria where PpIX is synthesized [13]. Several papers have presented results that indicate the important role of overexpressed ABCB6 in the elevated concentration of PpIX after 5-ALA administration [14,15]. Another type of ATP-binding cassette transporter is ABCB10, which is responsible for the production or efflux of 5-ALA from the mitochondria and plays a crucial role in the heme synthesis pathway [16]. ABCB10 also forms a complex with mitoferrin1 (MFNR1) and ferrochelatase (FECH), which intensifies the production of heme [17]. Another ATP-binding cassette transporter, which was the subject of the research, was ABCG2. This molecule is responsible for extracting PpIX from the mitochondria [18]. An elevated level of ABCG2 is connected with an insufficient PDT effect after 5-ALA administration. Therefore, many studies have described the inhibition of ABCG2 as a strategy for increasing the PpIX level in a cell [19]. Another important molecule that is involved in heme synthesis is MFNR1, which is responsible for mitochondrial iron delivery [20]. Silencing MFNR1 increased the PpIX levels in healthy mouse fibroblasts.

## 2. Results and Discussion

Using iron chelators in ALA-PDT to increase the efficiency of this therapy is not new. However, the number of chelators that can be used in ALA-PDT is limited and their mechanism of action is poorly understood. In our previous work, we investigated the ability of novel iron chelators from the TSC group to increase the PpIX accumulation on different cell lines with the heterogeneous expression of heme biosynthesis and degradation pathway genes. Of the TSC derivatives that were tested, derivative **2** emerged as the most promising in terms of its applicability in ALA-PDT because it increased the PpIX levels in most of the tested cell lines, did not exhibit any antiproliferative properties [8] and had good iron ion chelating characteristics [21].

**Figure 2:**
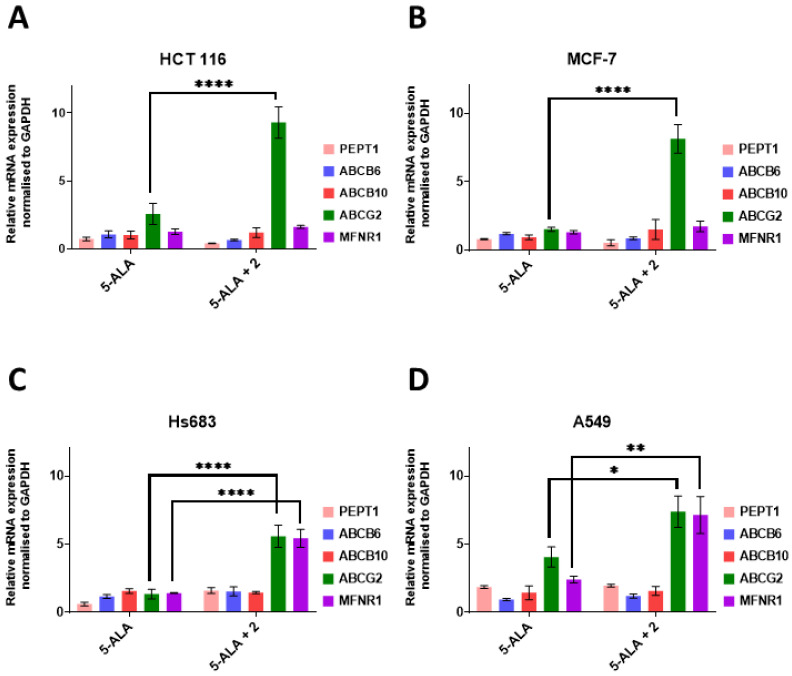
The expression of the genes that are associated with the transport of 5-ALA and heme after treatment with 5-ALA and a combination of 5-ALA with TSC derivative **2**. (**A**)—MCF-7, (**B**)—HCT 116, (**C**)—Hs683 and (**D**)—A549 cell lines. PEPT1—peptide transporter 1; ABCB6—ATP-binding cassette B6; ABCB10—ATP-binding cassette B10; ABCG2—ATP-binding cassette G2; MFRN1—mitoferrin 1. Data analysis was performed using a two-way ANOVA with Tukey’s post-hoc test: * *p* < 0.05, ** *p* < 0.005, **** *p* < 0.0001.

In this work, we continued our investigation of using iron chelators from the TSC group as an ALA-PDT enhancer, and focused on selected transporters that are involved in the flux of the heme biosynthesis pathway intermediates as well as on the efficacy of ALA-PDT in combination with TSC. The expression of these transporters is one of the factors that limits the efficacy of ALA-PDT. Of the selected transporters, only two resulted in statistically significant changes in the expression pattern after the 5-ALA treatment in combination with TSC derivative **2**. The first of these was ABCG2, which is one of the most studied transporters in this context and is involved in PpIX efflux from the cell, whose increased expression correlated with a low PpIX accumulation as well as a low PDT efficacy [14,22,23]. Statistically significant changes in the ABCG2 expression were observed in all of the tested cell lines after they had been with a combination of 5-ALA and TSC derivative **2** compared to the cells they had been treated with 5-ALA alone (Figure 2). The greatest changes in the expression of this transporter gene were observed for the MCF-7 cell line, in which there was a 5.3-fold increase in its expression after treatment with TSC derivative **2** combined with 5-ALA (Figure 2B), while for the HCT 116 and Hs683 cell lines, there was a 3.6- and 4.16-fold increase in its expression, respectively (Figure 2A,C). For the A549 cell line, the increase was the lowest, namely only 1.8-fold (Figure 2D). When the increase in the ABCG2 gene expression between cells that had been treated with 5-ALA and those that had been treated with the combination of TSC derivative **2** and 5-ALA were compared, it was found that the higher the ratio, the lower the PpIX accumulation, which was shown in our previous work (Table 1 [8]. This observation is consistent with the literature reports. For the exception was the A549 cell line, in which there was no significant accumulation of PpIX, and in which the increase in the ABCG2 expression was the smallest of the studied cell lines.

The second gene whose expression changed significantly for two cell lines after treatment with combination of 5-ALA and TSC derivative **2** compared to cells treated only with 5-ALA was mitoferin-1 (MFRN1), i.e., Hs683 (3.8-fold increase) and A549 (3.0-fold increase; Figure 2C,D). MFRN1, as was mentioned above, is responsible for the transport of iron ions into the mitochondria. Despite the paucity of literature on iron metabolism and ALA-PDT efficacy, it was shown that breast cancer cell lines that had a down-regulated expression of MFRN1 and MFRN2 had an increased accumulation of PpIX. In contrast, experiments using siRNA showed that silencing the mitoferrin genes resulted in an increased accumulation of PpIX in normal cells [20]. The increase in MFRN1 expression might explain the lack of PpIX accumulation on the A549 cell line after treatment with TSC derivative **2** that was observed in the previous work. In contrast, there was no such correlation for the Hs683 cell line.

**Figure 3:**
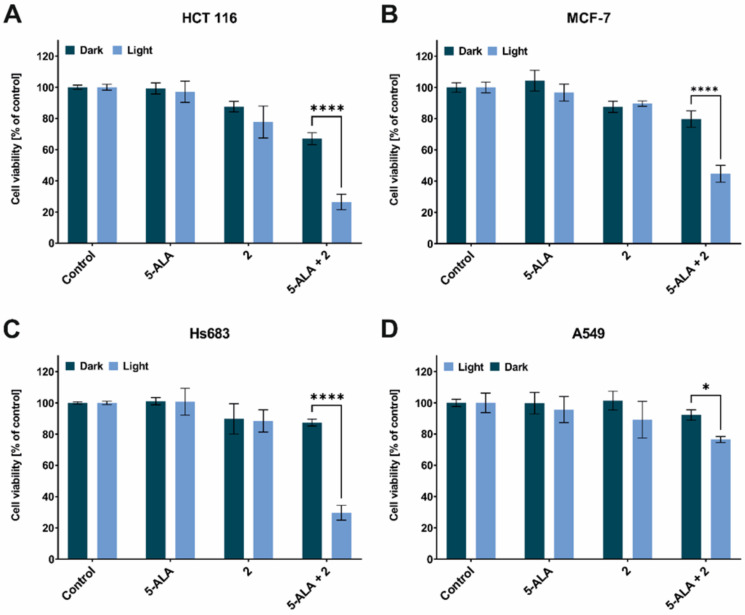
Phototoxic effect after red light irradiation at a dose of 12 J/cm^2^. (**A**)—MCF-7, (**B**)—HCT 116, (**C**)—Hs683 and (**D**)—A549 cell lines that had been treated with 5-ALA, TSC derivative **2** and their combination. Data analysis was performed using a two-way ANOVA with Tukey’s post-hoc test: * *p* < 0.05, **** *p* < 0.0001.

Based on the results presented above, and the available literature, it can be assumed that the phototoxic effect on the studied cell lines would be minor or that there would be no effect at all. However, the result of the irradiation of cells that had been treated with 5-ALA, TSC derivative **2** and their combination indicated that the viability decreased statistically significant (Figure 3). The strongest effect was observed for the HCT 116 and Hs683 cell lines (26% and 29% of the surviving fraction, respectively; Figure 3A,C). In the case of the MCF-7 line, the decrease of cell viability was 44% of the surviving fraction (Figure 3B). For the A549 line, the phototoxic effect was the weakest (76% of the surviving fraction; Figure 3D). The observed PDT effect for the HCT 116, MCF-7 and A549 cell lines correlated with the results for the PpIX accumulation because the higher the PpIX accumulation the stronger phototoxicity. Only for Hs683 cell line did the correlation between the PpIX accumulation and cell viability not correlate after irradiation, which is an open question for further research. The lack of a phototoxic effect after treatment with TSC derivative **2** alone is a desirable feature of a booster with potential use in ALA-PDT.

**Figure 4:**
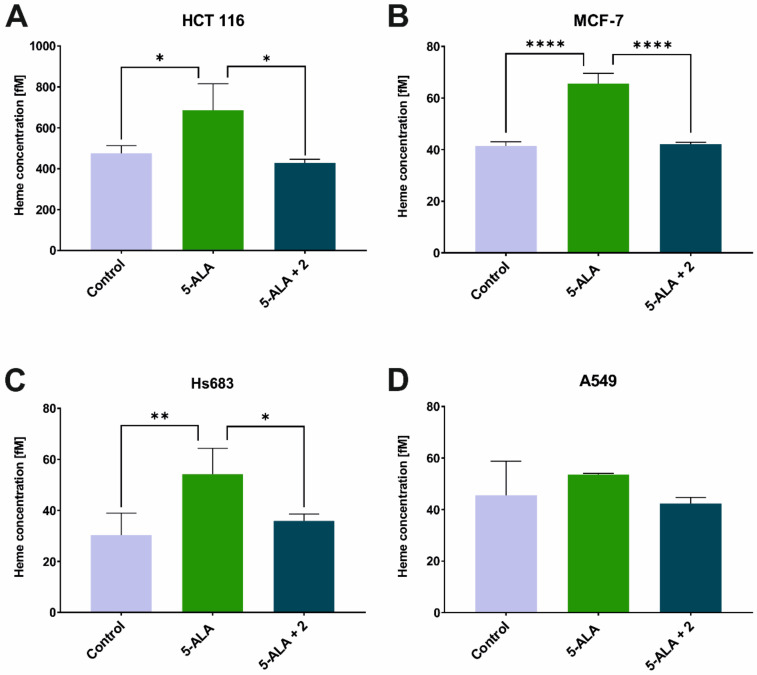
Free heme concentration after treatment of 5-ALA and combination of 5-ALA and TSC derivative **2**. (**A**)—MCF-7, (**B**)—HCT 116, (**C**)—Hs683 and (**D**)—A549 cell lines. Data analysis was performed using a one-way ANOVA with Tukey’s post-hoc test: * *p* < 0.05, ** *p* < 0.005, **** *p* < 0.0001.

Interestingly, there was no phototoxic effect on any of the tested cell lines that had been treated with 5-ALA alone. Most studies have shown that there is a stronger or weaker phototoxic effect during ALA-PDT. However, papers that show no or a weak phototoxic effect in ALA-PDT can also be found [24,25]. The increased expression of ABCG2, as was mentioned earlier, is responsible for the efflux of PpIX from the cell and in our opinion cannot explain the complete absence of a phototoxic effect only on the 5-ALA-treated cells. In order to explain this observation, we assumed that in the cells that had been treated with 5-ALA only, heme was still biosynthesized and thus the concentration of PpIX was insufficient to achieve a satisfactory therapeutic effect. Our results showed that the concentration of free heme significantly increased in the cells that had been treated with 5-ALA alone compared to the control and that when a combination of TSC derivative **2** with 5-ALA was applied, the concentration of heme decreased to a level in the HCT 116, MCF-7 and Hs683 cell lines that was similar to the control (Figure 4A–C). In the case of the A549 cell line, the level of free heme was not statistically significantly different after treatment with the tested compound combinations (Figure 4D). These observations in the 5-ALA-treated cells are not surprising because by exogenously providing 5-ALA, the regulatory system of heme biosynthesis through a negative feedback loop is overcome. In addition, the lack of iron deprivation permits the conversion of PpIX into heme. In the previous work, we showed that on MCF-7 and HCT 116 cell lines, the HO-1 expression levels were significantly elevated after treatment with 5-ALA alone and that they decreased after treatment with TSC derivative **2** in combination with 5-ALA [8]. This enzyme is, among others, responsible for the degradation of heme in cells [26] and its expression pattern correlates with the results that were obtained from the heme concentration study. On the other hand, one of the heme degradation products, i.e., biliverdin, acts as an antioxidant, which may explain the lack of a PDT effect on the 5-ALA-treated cell lines [27,28]. In contrast, for the Hs683 cell line, as was shown in a previous paper, the HO-1 levels increased after treatment with a combination of 5-ALA and TSC derivative **2**, which stands in the opposition to the facts that are presented above and yet, this line exhibited a significant decrease in cell viability after irradiation. For the A549 line, no changes were observed in the HO-1 gene expression or in the free heme concentration [8].

## 3. Materials and Methods

### 3.1. Cell Lines and Cell Culture

The HCT 116 (colon cancer), MCF-7 (breast cancer) and A549 (lung cancer) cell lines were purchased from the American Type Culture Collection (ATCC; Manassas, VA, USA), while the Hs683 (glioma) cell line was kindly provided by Prof. Gabriela Kramer-Marek from the Institute of Cancer Research (London, UK). All of the cell lines were cultured in Dulbecco’s Modified Eagle’s Medium (DMEM; Sigma-Aldrich; St. Louis, MO, USA), which had been supplemented with 12% foetal bovine serum (FBS; Sigma-Aldrich; St. Louis, MO, USA) and an antibiotic blend (1% *v/v* of penicillin and streptomycin; Gibco; Grand Island, NY, USA) in 75 cm^2^ flasks (Nunc, Sigma-Aldrich; St. Louis, MO, USA). The cells were cultured in standard conditions of 37 °C in a humidified atmosphere at 5% CO_2_. All of the cell lines were screened for Mycoplasma contamination.

### 3.2. Transporter Genes Expression

Cells were seeded in 3-cm plastic Petri dishes (Nunc, Sigma-Aldrich; St. Louis, MO, USA) at a density of 4 × 10^5^ cells per dish and incubated for 24 h at 37 °C. Then, the old medium was removed and replaced with solutions of 5-ALA (1 mM), derivative **2** (25 µM) and a combination of 5-ALA and derivative **2** in a manner in which their final concentrations matched the concentrations of 5-ALA and derivative **2** alone. After another 24 h of incubation, the total RNA was isolated from the cells with TRIzol Reagent (Invitrogen; Carlsbad, CA, USA) according to the manufacturer’s protocol. Reverse transcription was performed on 2 µg total RNA using a GoScript™ Reverse Transcriptase kit (Promega; Madison, WI, USA) and Oligo(dT)_23_ Primers (Sigma-Aldrich; St. Louis, MO, USA). Quantitative RT-PCR was performed in a CTX96 Touch™ Real-Time PCR Detection System (Biorad; Hercules, CA, USA) using SYBR^®^ Green Master Mix (Biorad; Hercules, CA, USA), the appropriate primer pairs and a cDNA template. The primers that were used in this study were designed using Primer 3 online software (Table 2) and synthesized by Sigma-Aldrich (Sigma-Aldrich; St. Louis, MO, USA). The data was analyzed based on a comparison of the expression of the target gene to a reference gene, GAPDH, using the 2^−ΔΔCT^ (Livak and Schmittgen) method. The experiments were repeated at least three times in triplicate.

### 3.3. Heme Concentration Measurement

The cells were seeded at a density 1 × 10^6^ cells/dish for 24 h at 37 °C. Next, 5-ALA (1 mM), TSC derivative **2** (25 µM) and a combination of 5-ALA and derivative **2** were added and incubated for 24 h in the dark at 37 °C. After incubation, the cells were washed with PBS and lysed with a 1% Triton X-100 solution in PBS. The lysates were then sonicated and centrifuged at 10,000× *g* for 10 min. The free heme concentration was measured using a Hemin Assay Kit (Abcam; Cambridge, UK) according to the manufacturer’s protocol. The heme concentration was calculated from the calibration curve of three independent experiments.

### 3.4. Phototoxic Effect

The cells were seeded into a 96-well plate at a density of 11,000 cells/well for 24 h at 37 °C. Then, the medium was removed and 5-ALA (1 mM), derivative **2** (25 µM) and a combination of 5-ALA and derivative **2** were added and incubated for 24 h in the dark at 37 °C. After the incubation, the cells were washed three times with a medium without phenol red and then irradiated or with an LED red light (634 ± 5 nm) at a dose 12 J/cm^2^. The cells were further incubated in the dark under 5% CO_2_ at 37 °C for 24 h. After irradiation, the cell viability was determined using an MTS assay. Briefly, the medium was removed and 100 µL of DMEM without phenol red and 20 µL of CellTiter 96^®^ AQueous One Solution (Promega; Madison, WI, USA) was added and then incubated for approximately 1 h at 37 °C. Simultaneously, the same experiment was performed with irradiation step being omitted in order to show dark cytotoxicity. The results are presented as the mean ± standard deviation of three independent experiments and the cell viability was calculated using the GraphPad Prism 5 software (GraphPad Software Inc.; San Diego, CA, USA).

### 3.5. Statistical Analysis

All of the experiments were repeated three times in independent experiments and the results are presented as the mean ± standard deviation. All of the data were analyzed using GraphPad Prism 5 software (GraphPad Software Inc.; San Diego, CA, USA). A two-way ANOVA followed by Tukey’s test was used to examine the significance of any differences between more than two groups. A two-tailed *p* value of 0.05 or less was considered significant.

## 4. Conclusions

In conclusion, the use of iron chelators from the thiosemicarbazone group as enhancers is a promising approach for improving the efficiency of ALA-PDT. The TSC derivative **2** is particularly interesting in this case due to its strong photocytotoxic effect, which was obtained on most of the tested cell lines in combination with 5-ALA. Despite the significant increases in ABCG2 gene expression, the observed therapeutic effect on most of the cell lines that were included in this work was substantial. This indicates that TSC derivative **2** is an excellent candidate for increasing the efficiency of ALA-PDT. We also showed that the expression level of the ABCG2 gene correlated with the phototoxic effect for most of the cell lines. The absence of this effect for the A549 lineage may be due to the increased expression of MFNR1. Surprisingly, this study does not explain the strong phototoxic effect that was observed for the Hs683 line and the reasons for this remain an open question. Because of the still insufficient knowledge about the mechanisms of ALA-PDT therapy, the results presented here are a necessary addition to fill in the existing gaps. At the same time, our study still remains an interesting area for further research.

## Figures and Tables

**Table 1:** Summary of results from previous work [8].

Cell line	Increase in fluorescence of PpIX relative to 5-ALA after treatment with TSC derivative **2** in combination with 5-ALA.	Expression of mRNA after treatment with TSC derivative **2** in combination with 5-ALA relative to 5-ALA.	
		FECH	HO-1
HCT 116	4.2-fold change	Down-regulated	Down-regulated
MCF-7	1.6-fold change	Non-significant changes	Down-regulated
Hs683	1.7-fold change	Up-regulated	Up-regulated
A549	Non-significant changes	Non-significant changes	Non-significant changes

**Table 2:** Primers used in this study.

Gene Name	Sequence 5′–3′ (F-Forward, R-Reverse)	
PEPT1	F	AGGCAACAACTATGTCCGGG
	R	CACAGCATCGAAGATCGGGA
ABCB6	F	CTGCGGTATGTGGTCTCTGG
	R	CCAGGTAGACTGTTGGGCTG
ABCB10	F	GTACGGGTCGCACGCA
	R	GTGAACGGCGATAGGGACC
ABCG2	F	GCACAGGAAGTTTACGCACAG
	R	AAGGGGCTAGAAGAAGGGGG
MFRN1	F	ACTCGGTGAAGACACGAATGC
	R	CAGCTATCCCGTTGGCTAGG
GAPDH	F	GAGTCAACGGATTTGGTCGTA
	R	GCCCCACTTGATTTTGGAG

## Data Availability

Not applicable.
